# Long Term Follow-Up of Stereotactic Body Radiation Therapy for Refractory Ventricular Tachycardia in Advanced Heart Failure Patients

**DOI:** 10.3389/fcvm.2022.849113

**Published:** 2022-04-29

**Authors:** John Wight, Thomas Bigham, Arielle Schwartz, Arslan T. Zahid, Neal Bhatia, Soroosh Kiani, Anand Shah, Stacy Westerman, Kristin Higgins, Michael S. Lloyd

**Affiliations:** ^1^School of Medicine, Emory University, Atlanta, GA, United States; ^2^UChicago Medicine, Chicago, IL, United States; ^3^Section of Cardiac Electrophysiology, Emory University, Atlanta, GA, United States; ^4^Department of Radiation Oncology, Emory University, Atlanta, GA, United States

**Keywords:** stereotactic body radiation therapy, refractory ventricular tachycardia, advanced heart failure, ventricular tachycardia ablation, ventricular tachycardia storm

## Abstract

**Background:**

Initial studies of stereotactic body radiation therapy (SBRT) for refractory ventricular tachycardia (VT) have demonstrated impressive efficacy. Follow-up analyses have found mixed results and the role of SBRT for refractory VT remains unclear. We performed palliative, cardiac radio ablation in patients with ventricular tachycardia refractory to ablation and medical management.

**Methods:**

Arrhythmogenic regions were targeted by combining computed tomography imaging with electrophysiologic mapping with collaboration from a radiation oncologist, electrophysiologist and cardiac imaging specialist. Patients were treated with a single fraction 25 Gy. Total durations of VT, the quantity of antitachycardia pacing (ATP) and shocks before and after treatment as recorded by implantable cardioverter-defibrillators (ICDs) were analyzed. Follow-up extended until most recent device interrogation unless transplant, death or repeat ablation occurred sooner.

**Results:**

Fourteen patients (age 50–78, four females) were treated and had an average of two prior ablations. Nine had ACC/AHA Stage D heart failure and three had left ventricular assist devices (LVAD). Two patients died shortly after SBRT, one received a prompt heart transplant and another had significant VT durations in the following months that were inaccurately recorded by their device. Ten of the 14 patients remained with adequate data post SBRT for analysis with an average follow-up duration of 216 days. Seven of those 10 patients had a decrease in VT post SBRT. Comparing the 90 days before treatment to cumulative follow-up, patients had a 59% reduction in VT, 39% reduction in ATP and a 60% reduction in shocks. Four patients received repeat ablation following SBRT. Pneumonitis was the only complication, occurring in four of the fourteen patients.

**Conclusion:**

SBRT may have value in advanced heart failure patients with refractory VT acutely but the utility over long-term follow-up appears modest. Prospective randomized data is needed to better clarify the role of SBRT in managing refractory VT.

## Introduction

SBRT (stereotactic body radiation therapy) has emerged as an experimental treatment for refractory ventricular tachycardia (VT) in recent years. Conventional ablations, while effective, have high rates of long term recurrence of VT and are unable to easily access deeper swaths of myocardium that frequently contribute to ablation failure (1–3). SBRT holds the potential advantage of reaching those deeper, larger portions of myocardium and is non-invasive (4).

Initial cases, case series and a prospective analysis have shown very impressive durable success in treating previously refractory VT with SBRT (5–7). Follow-up studies have been mixed but notably, the methods are quite heterogeneous and the role of SBRT in refractory VT treatment remains under investigation (7–12). Most prior studies have excluded advanced heart failure patients who make up an important subset of patients who have high burdens of VT/VF and have high mortality (13). Our prior case series of 10 initial patients treated with palliative SBRT for refractory VT demonstrated reasonable effectiveness in acutely reducing burdens of refractory VT/VF (8).

Here we report our cumulative experience with all follow-up to date with palliative SBRT treatment of advanced heart failure patients with refractory VT.

## Materials and Methods

Patients reported in this retrospective analysis received treatment under the compassionate use mechanism under the direction and approval of the Emory University Institutional Review Board. Methods were the same as reported in our prior retrospective analysis (8). All Patients considered for SBRT were required to have failed antiarrhythmic drugs, failed at least one RF ablation (or be inappropriate for RF ablation), or failed one adjunctive therapy such as mechanical support or sympathetic blockade, with failure defined as recurrent VT after intervention. Patients were required to provide consult with a radiation oncologist.

Wearable multielectrode vest technology with computed tomography (CT) registration (Cardio insight, Medtronic Corp., Minneapolis, MN) was not used in this cohort due to logistical constraints of this system applied to critically ill patients. All patients underwent at least one 3-dimensional imaging study and one electrophysiology study with electroanatomic mapping to identify the treatment target. Antiarrhythmic drug regimens were not altered after SBRT treatment, except for conversion to oral therapy.

The details of ablation modalities used and the general characteristics of ventricular arrhythmias of our cohort before SBRT are listed in Table 1.

**TABLE 1:** Characteristics of 14 patients undergoing SBRT.

Patient	Age	Gender	Diagnosis	Prior Ablations	Endo and/or epi	AAD Before	AAD After	Adjuncts	Stage D HF
1	53	F	NICM	1	Endo only	Amio 0.5 mg/min Sotalol 80 mg BID	Lido 0.5 mg/min	LVAD	Yes
2	55	M	ICM	4	Endo only	Carvedilol 25 mg BID, Amio 400 mg QD	Carvedilol 25 mg BID, Amio 400 mg QD, Mex 150 mg TID		Yes
3	65	M	NICM	2	Endo only	Sotalol 80 mg BID, metoprolol 50 mg QD	Sotalol 80 mg BID, metoprolol 50 mg QD		No
4	51	M	NICM	3	Endo/epi	Amio 400 mg QD, mex 150 mg BID, Phenytoin 200 mg BID	Amio 400 mg BID, mex 150 mg TID, Carvedilol 50 mg BID	Symp, LVAD	Yes
5	50	F	ICM	1	Endo only	Amio 400 mg BID, lido 1 mg/min.	Amio 400 mg BID	Symp	Yes
6	58	F	NICM, sarcoid	2	Endo/epi	Sotalol 120 mg BID, carvedilol 12.5 mg BID	Sotalol 120 mg BID, carvedilol 12.5 mg BID		No
7	78	M	ICM	1	Endo only	Amio 400 mg QD, Carvedilol 6.25 mg BID	Amiodarone 200 mg QD, Carvedilol 6.25 mg BID		No
8	70	M	ICM	1	Endo only	Metoprolol 12.5 mg QD, mex 250 mg Q8hr	Mex 250 mg Q8hr	IABP	Yes
9	57	M	NICM, myocarditis	5	Endo/epi	Sotalol 120 mg BID, Metoprolol 75 mg BID Lido 0.5 mg/min	Dofetilide 500 mg BID, Metoprolol 75 mg BID Mexilitine 150 mg Q8hr		No
10	61	M	ICM	2	Endo only	Amio 400 mg BID, Metoprolol 25 mg BID, mex 150 mg Q8hr	Amio 400 mg BID, Metoprolol 50 mg BID, mex 150 mg Q8hr	Symp	Yes
11	67	M	NICM	1	Endo only	Amio 400 mg QD, mex 150 mg TID	Amio 400 mg QD, mex 150 mg TID	LVAD	Yes
12	60	F	NICM	1	Endo only	Amio 1 mg/min, lido 1 mg/min	Amio 1 mg/min, lido 1 mg/min		Yes
13	66	M	NICM	1	Endo/epi	Amio 200 mg BID, Carvedilol 6.25 mg BID	Amio 200 mg BID, Carvedilol 6.25 mg BID		No
14	59	M	NICM, Sarcoid	0		Amio 400 mg QD, mex 150 mg TID	Amio 400 mg QD, mex 150 mg TID, Metoprolol 12.5 mg QD		Yes
*Net*	*61* + */7*	*10/14 M*	*5/14 ICM* *9/14 NICM*	*1.8* ± *1.1*		*2.2* ± *0.4*	*2.1* ± *0.7*	*6/14*	*9/14*

*AAD, antiarrhythmic drug; AA, after at 1 month following SBRT or closest other follow up. Amio, amiodarone; IABP, intra-aortic balloon pump; ICM, ischemic cardiomyopathy; Immunorx, immunotherapy; LVAD, left ventricular assist device; Lido, lidocaine; Mex, mexiletine; NICM, non-ischemic cardiomyopathy; Symp, sympathectomy or sympathetic blockade; Endo, endocardial; epi, epicardial.*

All but one patient had previously undergone extensive ablation procedures. Endocardial and epicardial voltage maps were obtained, with published designations of “scar” (0.5 mV bipolar), “transition zone” (0.5–1.5 mV bipolar), and “healthy” (>1.5 mV) being used. Epicardial voltage maps and use of unipolar mapping varied. Treatment strategies for each patient focused on scar homogenization, with use of entrainment for clinically tolerated VTs and pace-mapping as adjuncts. Powers ranged from 35 to 50 W using irrigated RF systems. Two types of mapping systems were used (EnSite Precision, Abbott, Abbott Park, IL; or CARTO, Biosense Webster, Diamond Bar, CA). Ablation catheters, mapping catheters, and intracardiac ultrasound systems included current-generation systems available in the United States. Definitions of inducibility and non-inducibility were determined by programmed ventricular stimulation that was performed before ablation and at end of the procedure using up to 3 extrastimuli to refractoriness or a coupling interval ≥ 200 ms.

Target zones for SBRT were chosen by 3-dimensional imaging and electroanatomically derived substrate, in addition to the recurrent VT morphology and comparison to remaining inducible VTs postablation. Of note, target planning for those patients with LVAD were performed in a manner similar to those without, and no clinically actionable changes were observed in LVAD pump speed, flow, pulsatility index, or power.

### Radiation Treatment

Before SBRT treatment, all patients underwent CT simulation. Rigid immobilization was used in a fashion consistent with SBRT treatment for lung cancers. All patients were simulated with administration of intravenous contrast if the estimated glomerular filtration rate was in the appropriate range. Axial images (1-mm slices) were obtained. A 4-dimensional CT was also obtained to assess target motion. For patients requiring intensive care unit care, continuous cardiac monitoring with telemetry was performed throughout radiation planning and treatment procedures.

For cardiac SBRT, structures at risk include the skin, spinal cord, lung, esophagus, rib, airway, and gastrointestinal organs, including the stomach and small bowel (particularly for targets located in the apex of the heart). Dose constraints for single-fraction SBRT for these normal organs were adopted from the TG101, a task force consensus statement regarding dose constraints and technical specifications for SBRT treatment constraints, with maximum point dose (MPD) for skin, 26 Gy; rib, 30 Gy; main bronchus, 20 Gy; spinal cord, 14 Gy; stomach, 12.4 Gy; duodenum, 12.4 Gy; esophagus, 15.4 Gy; and lungs at least 1,500 cc, < 7 Gy (14).

Planning target volumes (PTVs) were designed for all patients in collaboration with a team of specialists. Myocardial scar was identified on imaging studies and pretreatment electroanatomic mapping. Based on consensus, treatment areas were chosen as regions of scar identified as the source of the exit site of clinical VTs. The clinical target volume varied according to patient characteristics: in those with numerous exist sites, planned targets as determined in collaborative planning meetings encompassed all or most of identified substrate, while those with a single exit site, or those with very large scar burdens, had targets restricted to areas felt to be critical to arrhythmogenesis as determined by the details of prior electroanatomic mapping and EP study.

The region of myocardial scar was contoured in Eclipse treatment planning software (Varian, Palo Alto, CA), and PTV was created by expanding this region of scar by 1–5 mm. In order to ensure accuracy is maintained during transfer of the target into the CT space, an experienced operator who was both a clinician and software engineer created a MATLAB application that read the relevant information and plotted it in 3D for visualization and to compare it with medical reports. In a process similar to prior studies, this format was then manually validated using an interface in MATLAB to manually align the electroanatomic mapping output to the planning CT space (15, 16). Once the alignment was confirmed, a code converted this spatial information into a binary mask that could then be imported into a clinical viewing system where it was reviewed, again, visually to be in relation to the plan and other structures by this experienced operator. Of note, our team compared this approach with an automated registration for our cases, however after trying different approaches it was concluded than an expert review and frequent visual validation of the results was more practical than an automated registration. For automated registration to work, it has to match some anatomy or voxel values that are common in both images to be aligned, which was difficult to accomplish in our cases since the information from the electroanatomic mapping systems did not necessarily have an anatomic equivalent on the CT. The prescribed dose was 25 Gy in a single fraction. Volumetric modulated arc treatment was used for every patient. Radiation dosimetry mandated that 95% of the PTV received the prescription dose of 25 Gy, and heterogeneity of dose within the PTV ranged from 110 to 140% of the prescription dose. SBRT treatment was delivered on Varian Tru-beam linear accelerators. To ensure accurate target localization, KV images were taken of the patient in the treatment position and adjustments made from bony anatomic landmarks. A cone beam CT was then obtained and matched to bony anatomy. Further refinements were then made by the treating physician. At times, ICD leads were helpful landmarks located in the vicinity of the target and could aid in target localization.

### Statistical Analysis

Data analysis was stopped as of September 2021. At that time transplant, death or repeat ablation had occurred in the entire cohort. ICD detections and therapies were held constant pretreatment and posttreatment. Patients from our prior analysis were included and followed through their complete course after SRBT (8). The total seconds of detected VT or VF, total ICD shocks, and total antitachycardia pacing (ATP) sequences were tabulated for up to 3 months pretreatment and compared to posttreatment follow-up which extended until death, transplant or repeat ablation.

In order to normalize data due to the variable times of follow-up, the total VT seconds, ATP therapies, and ICD shocks were normalized to frequency per month per patient. Total ventricular arrhythmia burden was defined as VT seconds/30 days, ATP sequences/30 days, and ICD shocks/30 days.

Data regarding follow-up changes in VT/VF/NSVT, ATP and ICD shocks was included only from patients with useable ICD interrogations following SBRT. Patients were followed through the duration of their charted follow-up to assess for adverse events even after an outcome such as that of transplant or repeat ablation occurred.

## Results

As shown in Table 1, of the 14 patients undergoing SBRT, 10 were male, nine carried a diagnosis of non-ischemic cardiomyopathy (NICM), and nine had ACC/AHA Stage D heart failure. Patients had an average of 1.8 prior ablations and were on an average of 2 AEDs before SBRT. Notably, 3 patients had left ventricular assist devices (LVADs) and 1 patient had an intra-aortic balloon pump (IABP) at the time of treatment. One patient (patient 4) received repeat SBRT. ICD data for that patient extended until repeat SBRT and there were no repeat interrogations following their second SBRT as the patient received transplant shortly thereafter. The individual SBRT treatment details and outcomes are listed in Table 2. Three patients did not have a follow-up device interrogation after SBRT. Another patient (patient 3) had very significant VT burden following SBRT but which was not accurately recorded by their device and consequently was not included in the cumulative percent change data. Of the 10 patients with ICD interrogation data after SBRT, follow-up extended an average of 216 days.

**TABLE 2:** SBRT treatment and outcomes.

Patient	Target location	Margins (mm)	Follow-up months	Decrease in VT/VF	Months to first treated episode	Outcome	Adverse events
1	LV	1	0.5	Unknown	NA	Transplant	Pneumonitis
2	Lateral Apical LV	3	1.6	No	1.5	Transplant, died after	
3	LV summit	2	10.7	No	4.2	Repeat ablation ×3 (endo, surgical, alcohol)	
4	RV freewall	1	3.9	No	2.1	Repeat SBRT, Transplant	Pneumonitis
5	LV apex septum	1	0.2	Unknown	NA	Hospice	
6	Basal septum, LV anteroapex	2	2.0	Yes	0.1	Repeat ablation	
7	Apex	1	9.1	Yes	No recurrence	Hospice	
8	Posterolateral LV	3	9.6	No	0.1	Hospice	Pneumonitis
9	Anterobasal	5	6.7	Yes	0.4	Transplant	
10	LV apex	1	5.8	Yes	3.5	Hospice	
11	Pericannula	2	7.0	Yes	2.6	Transplant	Possible Pneumonitis
12	Inferolateral LV	1	0.1	Unknown	NA	Died shortly after SBRT	
13	Inferolateral LV	2	24.0	Yes	8.6	Repeat ablation	
14	Anteroseptal, anterolateral LV	1	2.0	Yes	No recurrences	Hospice, fungal pneumonia	
*Net*		*1.9* ± *1.1*	*5.9*	*64% (7/11)*	*9 with treated recurrences avg 2.6 months after* *2 without recurrence* *3 without follow up data*	*5 transplant (1 later died)*, *7 hospice/death* *3 repeat catheter ablation* *1 repeat SBRT, (later transplanted)*	*29% (4/14) Pneumonitis*

**FIGURE 1:**
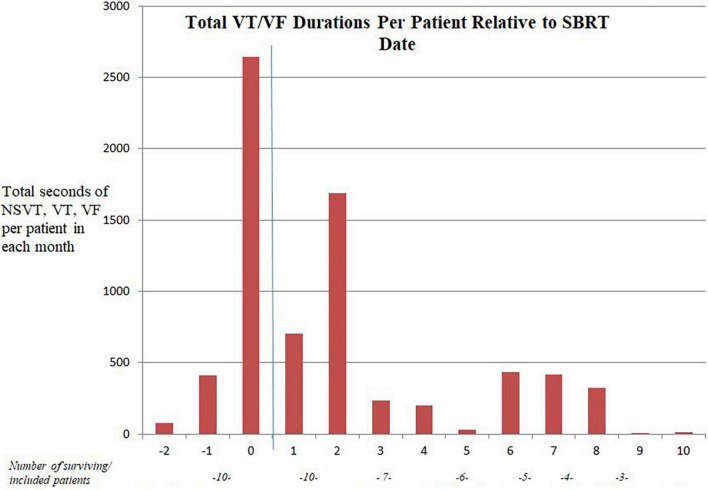
“Month” of follow-up relative to SBRT date. The blue line represents the SBRT date. Three patients had no follow-up ICD data after SBRT.

The collective data for the cohort of 14 patients is described in Table 3. Overall, on follow-up, there was a 59% reduction in VT/VF/NSVT, 39% reduction in ATP and 60% reduction in shocks over follow-up compared with the 3 months before SBRT. The change in VT/VF/NSVT per patient per month is shown in Figure 1. There was a substantial decrease in arrhythmia immediately following treatment though as described in other reports, the reduction VT/VF/NSVT was more pronounced after an initial washout period (6). Only six patients were still alive without transplant or repeat ablation after 5 months. Five of the original cohort of 14 patients went on to receive transplant.

**TABLE 3:** Cumulative follow-up data.

Cumulative follow-up data	
Reduction in VT, NSVT, VF*	59%
Reduction in ATP*	39%
Reduction in shocks*	60%
Mean time to first treated VT**	2.6 months
Transplants	5/14
Repeat ablations	4/14
Alive at 6 months	8/14
Alive at 12 months	7/14
Alive without transplant	3/14
Repeat ablations in patients alive without transplant	3/3
Complications (Pneumonitis)	4/14

*Only 10 of the original 14 patients had sufficient and valid follow-up ICD data to calculate percent changes in VT, ATP and shocks.

**Nine of 11 with follow up ICD data showing treated episodes.

Of the nine patients not receiving transplant, only three survived longer than 1 year after SBRT treatment, and all of those patients had eventual recurrent VT requiring repeat ablation. There were four patients in total who received repeat ablation following SBRT. Patient 3 had recurrent VT due to the same region near the LV summit and ultimately received additional ablations of the same area with alcohol, and a combined surgical/endovascular approach. Patient 4 had very significant burdens of recurrent VT following SBRT initially of the RV free wall and ultimately received repeat SBRT of the septum 3 months later before transplant. Patient 6 received repeat ablation after 2 months of effectively the same region over the basal septum and anterior wall. Patient 13 also had recurrent VT arising from a bordering region and received endovascular ablation over the lateral LV extending slightly anteriorly and posteriorly. All four of these patients who received repeat ablation had NICM. The mean time to repeat ablation was 10.2 months. Nine of the 11 patients with follow up ICD data after SBRT demonstrated recurrences in treated VT/VF with ATP or shocks with a mean time to first treated episode of 2.6 months.

The only adverse event related to SBRT occurring though the duration of charted follow-up was pneumonitis which likely occurred in four of the 14 patients. The details of each case of possible pneumonitis are described below.

Four months after SBRT treatment patient 1 presented to the hospital with cough and shortness of breath and was found to have opacities in the left lung. The patient was admitted due to her high-risk status with her recent transplant and immunosuppression but did not require supplemental oxygen. At the time, she was treated with 7 days of antibiotics for community acquired pneumonia but on review it was felt that there was a high likelihood this was radiation related pneumonitis.

Approximately 6 weeks following patient 4 receiving his first SBRT treatment, he was hospitalized for 1 week of cough without hypoxia with mild opacities on chest x ray which was treated with antibiotics, inhalers and a prednisone taper. He improved with these measures which on review was felt to likely represent radiation pneumonitis.

Patient 8 had a complicated course following SBRT, and 1 month later suffered a ground level fall and subdural hematoma which was managed conservatively. The patient had a history of both COPD and lung disease related to amiodarone toxicity with baseline 2–4L O2 requirements. 5 months after SBRT the patient presented initially with symptoms of heart failure but was found to have hypoxia requiring high flow nasal cannula which did not improve with diuresis. A CT chest demonstrated pneumonitis and after consultation from pulmonology, the patient with given intravenous methylprednisolone with improvement in his oxygenation to baseline over a few days. To complete treatment for possible pneumonitis, the patient was prescribed a 4-week taper of prednisone. That hospitalization was also complicated by worsening dysphagia and the patient transitioned to hospice care on discharge after many discussions with palliative care in line with his wishes.

Approximately 7 months following patient 11 receiving SBRT, while the patient was recovering from OHT in the intensive care unit he was noted to have a lingering oxygen requirement of 4L which did not improve with diuresis and a chest x ray showing bibasilar infiltrates. The ICU team was concerned for possible aspiration pneumonitis or pneumonia and treated the patient with 7 days of ceftriaxone. The patient was already on prednisone 10 mg daily for immunosuppression in relation to recent OHT but did not receive additional steroids at the time. His hypoxia improved over that 7-day treatment course with ceftriaxone, continued daily prednisone and oral diuretics.

Five of the 14 patients who underwent SBRT eventually received OHT. A description of the events leading to their transplant are overviewed below.

Patient 1 received OHT shortly after SBRT. She was listed for worsening heart failure symptoms despite LVAD, a chronic drive line infection and for recurrent VT. Her heart failure related symptoms included dyspnea on exertion, dizziness and generally did not correlate with her episodes of NSVT which were observed while the patient was hospitalized. The patient had a follow up trans thoracic echocardiogram completed shortly before her transplant which showed an ejection fraction of 10–15% which was unchanged from her prior before SBRT. The patient has done well following transplant.

Patient 2 received a OHT at an outside institution and presented to our institution specifically for consideration of SBRT. The patient received transplant due to a long history of significant ICM and VT. The patient died due to primary graft failure and several complications post-transplant. Due to his following for further treatment at an outside institution, we do not have access to the full details of his care after SBRT and OHT.

Patient 4 had a long-standing history of Stage D heart failure requiring LVAD. Two months after his initial SBRT he was admitted for symptoms of heart failure and later developed recurrent VT storm and received repeat SBRT. He was listed as 1A for heart transplant due to recurrent VT in the setting of prior LVAD implantation. The patient’s ejection fraction and left ventricular end systolic volume just prior to the first SBRT was 10% and 6.4 cm and shortly after the second SBRT 3 months later were effectively unchanged at 10% and 6.3 cm, respectively. The patient is doing well and following in transplant clinic.

Patient 9 initially improved after SBRT but had recurrent VT storm 6 months after treatment requiring CCU admission and intravenous lidocaine. In the context of his recurrent arrhythmia the patient also had progression of his heart failure and a significant reduction in ejection fraction. From just prior to SBRT his EF and left ventricular end systolic volume were 40% and 4.9 cm, respectively and 6 months following SBRT they were 20% and 5.9 cm, respectively. It is not possible to completely exclude SBRT as related to worsening of the patient’s heart failure, but his arrhythmia burden was felt to be the most likely causative factor. The patient has done well since transplant.

Patient 11 had an extensive history of NICM requiring LVAD prior to SBRT. Following SBRT, the patient initially had improvements in VT burdens but after 2 months began having VT episodes requiring ATP. Six months after SBRT the patient had increased burdens of VT and episodes of VF requiring shocks and the patient was placed on an amiodarone infusion and was upgraded to UNOS status 2 after which a suitable donor was identified approximately 1 week later. Just prior to SBRT, the patient’s ejection fraction was 10% with a left ventricular end systolic diameter of 6.0 cm with an LVAD in place. Six months later, the echocardiogram was effectively unchanged with an ejection fraction of 10% and a left ventricular end systolic diameter of 5.9 cm with the LVAD. Their post-operative course was complicated by pneumothorax requiring chest tube placement. The patient is now doing well and following in transplant clinic.

All patients in the cohort eventually reached an endpoint where they received transplant, died or received repeat ablation.

## Discussion

Our retrospective cohort of critically ill advanced heart failure patients was notable for a few findings. Our cohort of advanced heart failure patients which includes 8 ACC/AHA Class D patients, 3 patients with LVADs, and 1 with an IAPB is apparently more critically ill than any other published cohort to our knowledge. Our results again demonstrate that there is an immediate reduction in VT burden following treatment showing the potential utility of SBRT in the management of refractory VT even in critically ill advanced heart failure patients. This acute reduction in VT burden importantly helped bridge five patients to transplant.

While there was a significant acute reduction in VT burden, there were late recurrences requiring ablation in all patients surviving patients without transplant. Our total net reduction in VT is lower than reported in most other cohorts and clinical trials and there are a few differences in patients and methodology which could account for the discrepancy (6, 7, 9, 10). As noted previously, due to practical constraints and the critically ill state of many of these patients, wearable multielectrode vest technology was not used which could have better localized the arrhythmogenic origin. Of the four patients who received repeat ablation, one had VT mapped and ablated at different localization (the septum vs. the RV wall) for which initial use of the wearable multielectrode vest may have been important. This cohort was also more critically ill than others as previously mentioned and with more patients with NICM which also could account for some of the differences. Also of note, patient 12, died shortly after SBRT. Acute toxicity, when reported in prior series, with SBRT is generally mild, however, severe complications can occur. This particular patient had continued electrical storm and progressive hemodynamic collapse which we believe was the cause of death, but we cannot entirely exclude an adverse reaction to SBRT. Patient 9 had a significant decline in their EF following SBRT and ultimately required transplantation, and while this was more likely directly related to the significant burdens of VT, a direct radiation related toxicity cannot completely be excluded. Four other patient had clinical syndromes following SBRT possibly consistent with pneumonitis. Patient 11’s symptoms were slightly outside the expected timeframe for pneumonitis and had alternative explanations including possible aspiration but we did include this patient as a possible case of pneumonitis. The other patients fit well into the expected time frames and symptoms and were overall quite consistent with radiation pneumonitis (17).

Our study is additionally limited due to its small size and retrospective cohort design. Further in depth sub-group statistical analysis cannot reasonably be performed due to these limitations. Given the relatively short average follow-up duration we additionally cannot accurately assess the long term safety of this experimental therapy.

## Conclusion

SBRT may have value in advanced heart failure patients with refractory VT acutely and to aid bridging to transplant, but its utility over long-term follow-up appears modest. Prospective randomized data is needed to better clarify the role of SBRT in managing refractory VT.

## Data Availability Statement

The original contributions presented in the study are included in the article/supplementary material, further inquiries can be directed to the corresponding author/s.

## Ethics Statement

The studies involving human participants were reviewed and approved by the Emory University IRB. The patients/participants provided their written informed consent to participate in this study.

## Author Contributions

JW: data acquisition and manuscript writing. ASc and TB: data acquisition. AZ: editing and data presentation. NB, SK, ASh, SW, and KH: clinical care. ML: clinical care and editing. All authors contributed substantially to this work.

## Conflict of Interest

The authors declare that the research was conducted in the absence of any commercial or financial relationships that could be construed as a potential conflict of interest.

## Publisher’s Note

All claims expressed in this article are solely those of the authors and do not necessarily represent those of their affiliated organizations, or those of the publisher, the editors and the reviewers. Any product that may be evaluated in this article, or claim that may be made by its manufacturer, is not guaranteed or endorsed by the publisher.
